# A novel frameshift c.22_25dupGCAT mutation of the *NDP* gene in a Chinese infant with Norrie disease: A case report

**DOI:** 10.1097/MD.0000000000028523

**Published:** 2022-01-07

**Authors:** He Wang, Zeyuan Liu, Yuantao Zhou, Yuanyuan Ma, Dan Tao

**Affiliations:** a Department of Ophthalmology, Kunming Children's Hospital, Kunming, Yunnan, China; b Kunming Key Laboratory of Children Infection and Immunity, Yunnan Key Laboratory of Children's Major Disease Research, Yunnan Medical Center for Pediatric Diseases, Yunnan Institute of Pediatrics, Kunming Children's Hospital, Kunming, Yunnan, China.

**Keywords:** a novel mutation, *NDP* gene, next-generation sequencing, Norrie disease

## Abstract

**Rationale:**

Norrie disease (ND) is a rare X-linked recessive disease characterized by bilateral congenital blindness and auditory impairments. According to the previous studies, Norrin cystine knot growth factor (*NDP*) gene have been found to be responsible for ND. Herein, we report a case of ND with a novel mutation in *NDP* and elucidate the clinical and molecular characteristics of this patient.

**Patient concerns:**

A 2-month-old Chinese male infant presented with gray-white opacification in the bilateral cornea. Vitreous opacity and retinal detachment were observed on ocular ultrasound. Furthermore, a novel *de novo* hemizygous mutation (c.22_25dupGCAT, p.S9Cfs∗18) in exon 2 of the *NDP* gene was identified by next-generation sequencing. SWISS-MODEL predicted that the c.22_25dupGCAT mutation truncated the NDP protein.

**Diagnosis:**

Based on the above clinical and genetic evidence, this patient was eventually diagnosed with ND.

**Interventions:**

Currently, no clinical therapy is available for ND.

**Outcomes:**

In addition to the typical ocular symptoms, no other abnormalities were observed. The patient's vital signs remained stable and normal.

**Lesson:**

A novel causative mutation of *NDP* was identified using next-generation sequencing. Our report expands the pathogenic mutation spectrum of *NDP* and facilitates genetic counseling and prenatal diagnosis. Additionally, we emphasize the importance of molecular genetic testing in the diagnosis of ND.

## 1 Introduction

Norrie disease (ND, OMIM#310600) is a rare X-linked recessive disorder characterized by varying degrees of visual impairment and progressive hearing loss, with a prevalence of 1 per 100,000 people.^[1,2]^ Patients with ND usually present with bilateral congenital or early childhood blindness, among which 30% to 50% of these patients have progressive neurodevelopmental retardation.^[3]^ Meanwhile, there are multiple ocular manifestations, including corneal opacification, cataracts, microphthalmia, retinal detachment and dysplasia, iris hypoplasia, and vitreous hemorrhage. Progressive hearing loss can be found in approximately one-third of ND cases, and is usually evident during the second decade of life.^[4]^ With the development of numerous related studies, *the NDP* gene has been found to be the molecular basis of ND.

**Figure 1:**
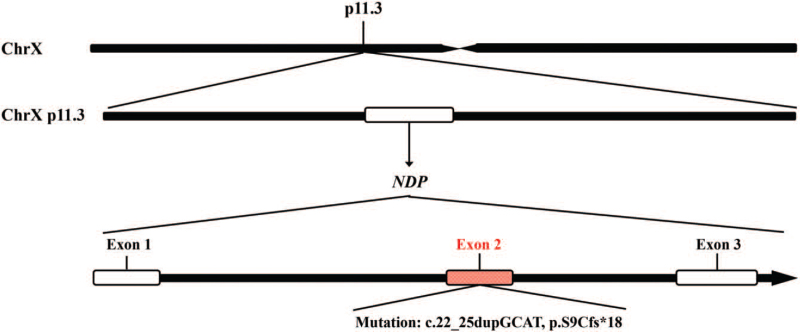
Schematic diagram of *NDP* gene structure, location and corresponding encoded NDP protein. *NDP* = Norrin cystine knot growth factor.

The Norrin cystine knot growth factor (*NDP*) gene is a 24,615-nt long gene located on chromosome Xp11.3, contains 3 exons (Fig. 1), and is widely expressed in several tissues, including the eye, ear, and brain.^[4]^ The *NDP-encoded* secretory protein Norrin consists of 133 amino acids, which mediates the activation of the Wnt/β-catenin pathway, thereby regulating cell division and differentiation, and plays a crucial role in the development of the retina, cochlea, and central nervous system.^[5–7]^ The variants in *NDP* have been found to be responsible for ND. In the clinical applications of gene sequencing, more than 160 mutations in *the NDP* gene have been described to date.^[8]^ Here, we report a rare case of ND in China that aimed to reveal a novel hemizygous mutation of *the NDP* gene that contributes to the development of ND in this patient, thus expanding the pathogenic mutation database of *NDP*. This study was approved by the ethics committee of Kunming Children's Hospital, and written informed consent was obtained from the parents of the patient for publication of this manuscript.

## 2 Case presentation

**Figure 2:**
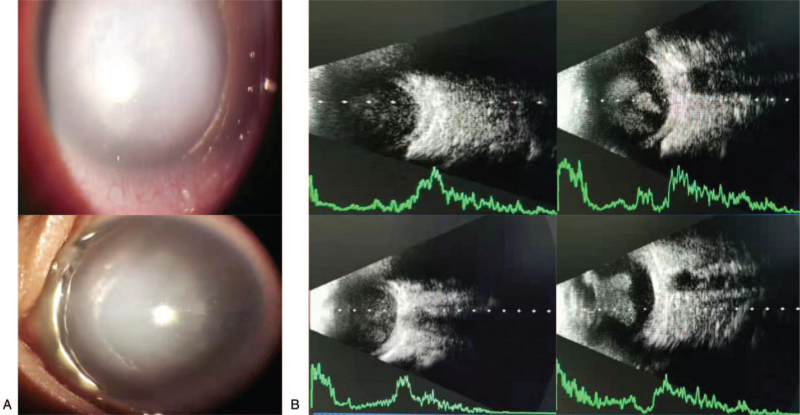
Ocular symptoms in the patient. (A) The result of slit lamp examination. (B) The result of ocular ultrasound.

A 2-month-old male patient with suspected ND was admitted to Kunming Children's Hospital. After admission, a detailed clinical evaluation and genetic testing were performed. Slit lamp examination revealed gray-white opacification in both corneas (Fig. 2A). Corneal opacity and edema, abnormal pupillary light reflex, and normal anterior chamber depth were observed via ocular ultrasound, which suggested vitreous opacity and retinal detachment (Fig. 2B). Additionally, neurodevelopment and hearing in this patient were also assessed, and no abnormalities were detected.

**Figure 3:**
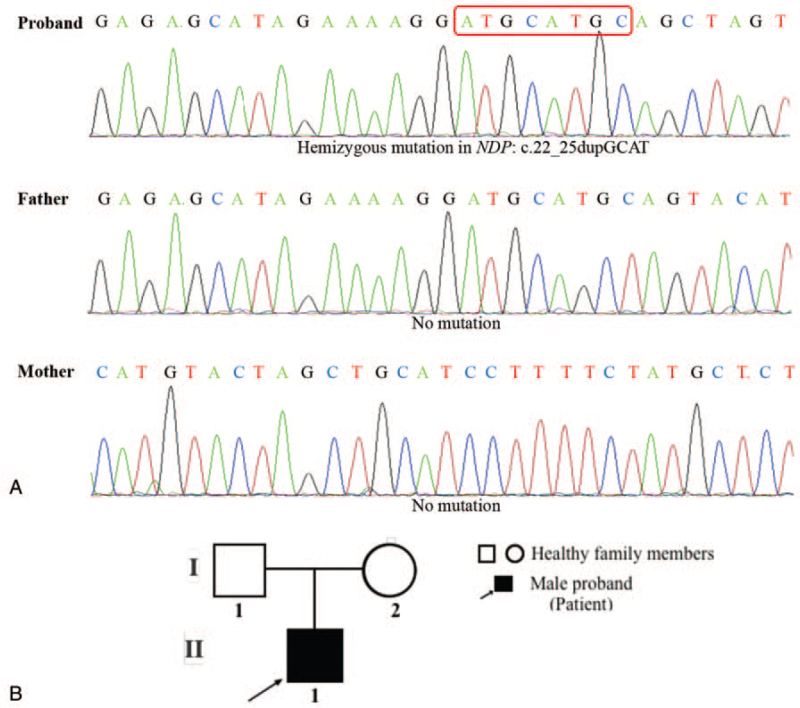
Mutation analysis of all subjects. (A) Reverse complementary DNA sequence acquired through high-throughput sequencing. A novel *de novo* hemizygous mutation of *NDP* gene in the proband was identified (c.22_25dupGCAT, p.S9Cfs∗18). The mutation was indicated by the red box. (B) Pedigree of the patient's family. The patient's parents are both healthy and no mutation was detected in them. *NDP* = Norrin cystine knot growth factor.

**Figure 4:**
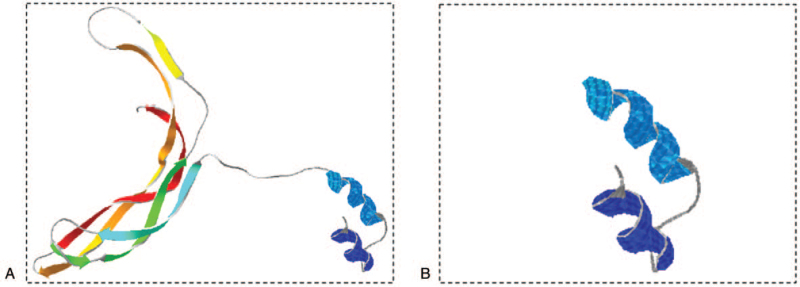
The 3D structural models of the wild type and mutant NDP protein. (A) Structural model of the wild NDP protein. (B) Structural model of the mutant NDP protein showed a truncation of NDP. *NDP* = Norrin cystine knot growth factor.

An investigation at the genetic level was performed using next-generation sequencing to identify the underlying causative mutation for the clinical phenotype in our patient. The results showed a novel hemizygous mutation in exon2 of *the NDP* gene of the proband (patient). The c.22_25dupGCAT mutation resulted in a premature termination codon at the position of 18 codons after mutation site p.9 (c.22_25dupGCAT, p.S9Cfs∗18) (Fig. 3). According to subsequent Sanger sequencing, this mutation was spontaneous due to the fact that the mutation was not found in his biological parents (Fig. 3). To the best of our knowledge, there have been no previous reports of the c.22_25dupGCAT mutation in *NDP,* and the frequency of this mutation in the normal population is unknown. This novel mutation was classified as pathogenic based on the guidelines of the American College of Medical Genetics and Genomics. Subsequently, SWISS-MODEL, an automated protein homology modelling server, was used to predict the structure of *the Mut-*NDP protein using the amino acid sequence obtained from sequencing. The three-dimensional structure model of *the Mut*-NDP protein revealed that the c.22_25dupGCAT mutation truncated the NDP protein (Fig. 4). Taking the above findings together, the *NDP* c.22_25dupGCAT mutation was predicted to be the genetic cause of the ND phenotype in this patient. Currently, no clinical therapy is available for ND. In addition to the typical ocular symptoms, no other abnormalities were observed. The patient's vital signs remained stable and normal.

## 3 Discussion

ND is inherited in an X-linked recessive manner and is mainly coupled with severe dual sensory impairment caused by *NDP* variants.^[9,10]^ Norrin, a secreted cystine knot growth factor encoded by *NDP*, was verified to function as the direct ligand of Frizzled-4 thus activating canonical Wnt signaling pathway through the interaction with Frizzled-4, lipoprotein receptor-related protein-5/6 as well as tetraspanin-12 and the defect of Wnt signal involved in a series of biological processes, such as angiogenesis.^[11,12]^ A study conducted by Richter et al^[13]^ revealed retinal vessel malformation and impaired formation of deep retinal capillaries in *NDP*-knockout mice and uncovered the necessity of *NDP* products for retinal angiogenesis and development. Similarly, Rehm et al^[14]^ found that the knockout mouse model with a deleted *NDP* had serious deafness, and the primary lesions were mapped to the stria vascularis in the cochlea, where nearly two-thirds of the vessels were lost. Their result proved that norrin plays a critical role in cochlear development and function. Consistent with the above research, transgenic expression of ectopic norrin protein was found to provide neurotrophic support and recover the generation of the vessel network within the retinal tissue in mice with *the NDP* mutation.^[15]^ Overall, norrin protein has been shown to be involved in the Wnt signaling cascade, thereby regulating angiogenesis and the development of the retina and cochlea. In our study, the proband carried a novel frameshift *NDP* mutation (c.22_25dupGCAT, p.S9Cfs∗18) and presented an ND phenotype.

ND presents as diverse clinical manifestations. In addition to the typical ophthalmic symptoms, some individuals with ND can exhibit various degrees of developmental delay, progressive cognitive impairment, and mental retardation, and a few patients even have more severe and complex clinical phenotypes, including seizures and growth failure.^[16]^ In our report, the patient only showed obvious ocular lesions, without other abnormalities. The clinical severity of ND may be associated with the type and location of *the NDP* mutations. Currently, more than 160 mutations in *NDP* have been reported, of which approximately 50% are missense mutations, approximately 26% are deletion mutations, and the remaining 24% include nonsense mutations, frameshift mutations, in-frame mutations, and splice mutations (http://www.hgmd.cf.ac.uk). 95% of these reported mutations are responsible for ND, and the remaining 5% are related to X-linked familial exudative vitreoretinopathy, Coats disease, and retinopathy of prematurity.^[17–19]^ However, the detailed genotype-phenotype relationship remains unclear. Even the same *NDP* mutation may cause diverse clinical phenotypes. For example, Zhang et al^[17]^ reported a heterozygous *NDP* c.-1_2delAAT mutation in a Chinese family and 2 male family members were eventually diagnosed with ND. It is interesting to note that mild intellectual disability was observed in 1 patient but not in another. The similar phenomenon was also described by Riveiro-Alvarez et al.^[3]^ In their study, 2 boys from the same family with an *NDP* c.529C>T mutation had different phenotypes. Specifically, the proband was blind at the age of 5, while his brother had normal visual acuity in his right eye. Furthermore, this c.529C>T mutation was reported in a French family that presented with a more severe ND phenotype.^[20]^ These studies suggest that some other unknown genetic, epigenetic, and environmental factors may play an essential role in disease phenotype and progression. Therefore, the application of high-throughput sequencing is necessary for the precise diagnosis of ND owing to the high clinical and genetic heterogeneity of this disease.

In summary, we identified a novel frameshift mutation in *NDP* in a Chinese infant and elucidated its detailed clinical characteristics. This novel *NDP* mutation was speculated to be the molecular pathological cause of the ND in our patient. Our study expands the mutation spectrum of *NDP*, further facilitates the understanding of the molecular mechanism of ND, and provides a basis for exploring the relationship between ND genotype and phenotype.

## Acknowledgments

We sincerely thank the patients and their family members for their participation and support.

## Author contributions

**Data curation:** He Wang, Zeyuan Liu.

**Formal analysis:** He Wang, Yuantao Zhou, Yuanyuan Ma.

**Investigation:** Zeyuan Liu.

**Methodology:** He Wang, Yuantao Zhou, Yuanyuan Ma.

**Project administration:** Dan Tao.

**Writing – original draft:** He Wang, Zeyuan Liu, Yuantao Zhou, Dan Tao.

**Writing – review & editing:** Dan Tao.

## References

[1] Norrie G. Causes of blindness in children. Acta Ophthalmol 1927;5:357–86.

[2] Rodríguez-Muñoz A García-García G Menor F Millán JM Tomás-Vila M Jaijo T. The importance of biochemical and genetic findings in the diagnosis of atypical Norrie disease. Clin Chem Lab Med 2018;56:229–35. 28742514 10.1515/cclm-2017-0226

[3] Riveiro-Alvarez R Trujillo-Tiebas MJ Gimenez-Pardo A. Genotype-phenotype variations in five Spanish families with Norrie disease or X-linked FEVR. Mol Vis 2005;11:705–12. 16163268

[4] Chen ZY Hendriks RW Jobling MA. Isolation and characterization of a candidate gene for Norrie disease. Nat Genet 1992;1:204–8. 1303236 10.1038/ng0692-204

[5] Shen G Ke J Wang Z. Structural basis of the Norrin-Frizzled 4 interaction. Cell Res 2015;25:1078–81. 26227961 10.1038/cr.2015.92 PMC4559814

[6] Xu Q Wang Y Dabdoub A. Vascular development in the retina and inner ear: control by Norrin and Frizzled-4, a high-affinity ligand-receptor pair. Cell 2004;116:883–95. 15035989 10.1016/s0092-8674(04)00216-8

[7] Warden SM Andreoli CM Mukai S. The Wnt signaling pathway in familial exudative vitreoretinopathy and Norrie disease. Semin Ophthalmol 2007;22:211–7. 18097984 10.1080/08820530701745124

[8] Jia LY Ma K. Novel Norrie disease gene mutations in Chinese patients with familial exudative vitreoretinopathy. BMC Ophthalmol 2021;21:84. 33588793 10.1186/s12886-021-01852-3 PMC7885586

[9] Saini JS Sharma A Pillai P Mohan K. Norries disease. Indian J Ophthalmol 1992;40:24–6. 1464451

[10] Warburg M. Norrie's disease--differential diagnosis and treatment. Acta Ophthalmol (Copenh) 1975;53:217–36. 808085 10.1111/j.1755-3768.1975.tb01156.x

[11] Chang TH Hsieh FL Zebisch M Harlos K Elegheert J Jones EY. Structure and functional properties of Norrin mimic Wnt for signalling with Frizzled4, Lrp5/6, and proteoglycan. Elife 2015;4:e06554. 10.7554/eLife.06554 PMC4497409 26158506

[12] Junge HJ Yang S Burton JB. TSPAN12 regulates retinal vascular development by promoting Norrin- but not Wnt-induced FZD4/beta-catenin signaling. Cell 2009;139:299–311. 19837033 10.1016/j.cell.2009.07.048

[13] Richter M Gottanka J May CA Welge-Lüssen U Berger W Lütjen-Drecoll E. Retinal vasculature changes in Norrie disease mice. Invest Ophthalmol Vis Sci 1998;39:2450–7. 9804153

[14] Rehm HL Zhang DS Brown MC. Vascular defects and sensorineural deafness in a mouse model of Norrie disease. J Neurosci 2002;22:4286–92. 12040033 10.1523/JNEUROSCI.22-11-04286.2002 PMC6758776

[15] Ohlmann A Scholz M Goldwich A. Ectopic norrin induces growth of ocular capillaries and restores normal retinal angiogenesis in Norrie disease mutant mice. J Neurosci 2005;25:1701–10. 15716406 10.1523/JNEUROSCI.4756-04.2005 PMC6725931

[16] Berger W van de Pol D Warburg M. Mutations in the candidate gene for Norrie disease. Hum Mol Genet 1992;1:461–5. 1307245 10.1093/hmg/1.7.461

[17] Zhang XY Jiang WY Chen LM Chen SQ. A novel Norrie disease pseudoglioma gene mutation, c.-1_2delAAT, responsible for Norrie disease in a Chinese family. Int J Ophthalmol 2013;6:739–43. 24392318 10.3980/j.issn.2222-3959.2013.06.01 PMC3874509

[18] Dickinson JL Sale MM Passmore A. Mutations in the NDP gene: contribution to Norrie disease, familial exudative vitreoretinopathy and retinopathy of prematurity. Clin Exp Ophthalmol 2006;34:682–8. 16970763 10.1111/j.1442-9071.2006.01314.x

[19] Black GC Perveen R Bonshek R. Coats’ disease of the retina (unilateral retinal telangiectasis) caused by somatic mutation in the NDP gene: a role for norrin in retinal angiogenesis. Hum Mol Genet 1999;8:2031–5. 10484772 10.1093/hmg/8.11.2031

[20] Royer G Hanein S Raclin V. NDP gene mutations in 14 French families with Norrie disease. Hum Mutat 2003;22:499. 14635119 10.1002/humu.9204

